# Molecular and Serological Tests for COVID-19. A Comparative Review of SARS-CoV-2 Coronavirus Laboratory and Point-of-Care Diagnostics

**DOI:** 10.3390/diagnostics10060434

**Published:** 2020-06-26

**Authors:** Robert Kubina, Arkadiusz Dziedzic

**Affiliations:** 1 Department of Pathology, Faculty of Pharmaceutical Sciences in Sosnowiec, Medical University of Silesia, 40-055 Katowice, Poland; 2 Department of Conservative Dentistry with Endodontics, Faculty of Medical Sciences in Zabrze, Medical University of Silesia, 40-055 Katowice, Poland; adziedzic@sum.edu.pl

**Keywords:** COVID-19, SARS-CoV-2, in vitro diagnostic tests, RT-qPCR, serology immunoassays

## Abstract

Validated and accurate laboratory testing for Severe Acute Respiratory Syndrome Coronavirus 2 (SARS-CoV-2) is a crucial part of the timely management of Coronavirus Disease 2019 (COVID-19) disease, supporting the clinical decision-making process for infection control at the healthcare level and detecting asymptomatic cases. This would facilitate an appropriate treatment, a prompt isolation and consequently deceleration of the pandemic. Various laboratory tests can identify the genetic material of SARS-CoV-2 that causes COVID-19 in specimens, or specific anti-viral antibodies in blood/serum. Due to the current pandemic situation, a development of point-of-care diagnostics (POCD) allows us to substantially accelerate taking clinical decisions and implement strategic planning at the national level of preventative measures. This review summarizes and compares the available POCD and those currently under development, including quantitative reverse transcription PCR (RT-qPCR), serology immunoassays (SIAs) and protein microarray method (PMM) designed for standard and rapid COVID-19 diagnosis.

## 1. Introduction

Coronavirus Disease 2019 (COVID-19), caused by the novel coronavirus Severe Acute Respiratory Syndrome Coronavirus 2 (SARS-CoV-2, formerly known as 2019-nCoV), appeared in China for the first time, and subsequently spread worldwide [1,2]. On 30 January 2020, the World Health Organization (WHO) officially announced the COVID-19 epidemics as a threat to public health internationally, and subsequently, in March 2020, the global situation escalated into the COVID-19 pandemic. Johns Hopkins University reported over 7,600,000 cases of infection and more than 427,000 deaths as of 13 June 2020 [3]. As a result of this rapidly progressing COVID-19 pandemic and the limited laboratory-based molecular testing capacities, new point-of-care (POC), scalable rapid diagnostic tests have been invented recently as easy-to-use tools to allow COVID-19 diagnostics outside of laboratory settings. What is more, the urgent need to multiply testing for COVID-19 has been clearly identified as an essential element of the anti-coronavirus strategy all over the world.

The diagnostic sensitivity, specificity and accuracy concerning suspected COVID-19 infection, based on controlled testing and performance data from clinical settings, is of substantial importance in the context of limiting the scope of coronavirus epidemics. Unreliable and unproved tests may not detect patients with active infection or may incorrectly indicate COVID-19-negative patients as positive, hampering healthcare efforts. The diagnostic laboratory and point-of-care tests (POCTs) used in order to detect SARS-CoV-2 are, first of all, reference tests based on molecular technique real-time quantitative reverse transcriptase polymerase chain reaction assay (RT-qPCR) as well as serological antibody-detecting and antigen-detecting tests, for auxiliary purposes. At present, only molecular quantitative reverse transcription PCR (RT-qPCR) testing of respiratory tract samples is the recommended method for the identification and laboratory confirmation of COVID-19 cases, as these methods were evaluated for their quality and safety through the World Health Organization (WHO) protocols [4,5]. On the other hand, based on current scientific evidence, WHO recommends the use of POC immunodiagnostic tests for research purposes and, at present, they should not be utilized in a clinical decision-making setting and in patient care until fully validated, with supporting data available. However, they can be useful in epidemiologic research or disease surveillance and further evolve as a critical step to develop COVID-19 vaccine in future. At the time of increased demand for hospital services, clinicians, governments and health services urgently need a fast, sensitive, but at the same time inexpensive diagnostic test, in order to rapidly manage patients, regarding admissions to hospitals meant for COVID-19 treatment. Therefore, the role of an approved and reliable diagnostic test in the COVID-19 care pathway is of the utmost importance.

As in the case of other infectious diseases, the RT-qPCR method, as well as serological tests, are suitable for the in vitro diagnostics (IVD) of patients suspected of being infected with SARS-CoV-2. Genetic assay based on the RT-qPCR technique, performed in real time, especially plays a role at the early stages of viral infection, when the virus multiplies quickly, as this technique enables direct detection of the pathogen’s genetic material. A different approach in diagnosing infection is represented by serological methods, based on detecting—in blood serum—of antibodies that act specifically against viral proteins, which are produced in response to SARS-CoV-2. Serological IVD tests detect antibodies which are responsible for neutralizing the virus; therefore, this implies that they are used when the immunological reaction against SARS-CoV-2 virus is already taking place. These IVD measures can determine how fast antibodies fighting the virus are produced, which may influence the identification of subjects who already developed immunity. The results of serological IVD tests should not be used as basis for diagnosing, ruling out infection with SARS-CoV-2, or informing about infection status [6].

**Figure 1:**
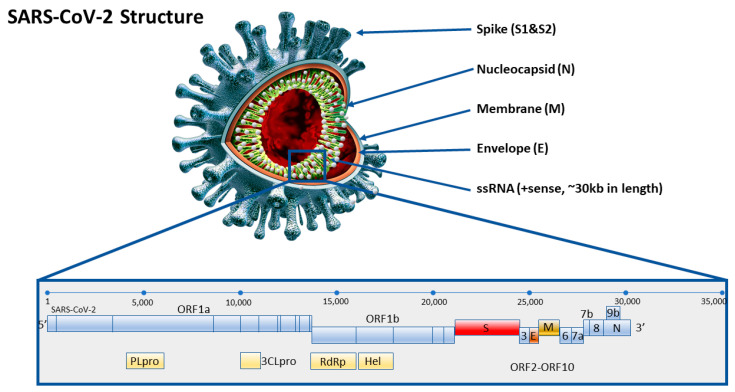
Real Time PCR COVID-19 genetic tests detecting ORF1ab, spike (S), envelope (E), or nucleocapsid (N) gene sequences of SARS-CoV-2 coronavirus (own interpretation, based on [8]).

The relatively quick discovery of the composition of the full genome of SARS-CoV-2 early during the epidemics made it easier to develop specific starters and normalized laboratory protocols for COVID-19. The protocol of the first RT-qPCR test, focused on RNA-dependent RNA polymerase (RdRp), envelope (E) and nucleocapsid (N) of SARS-CoV-2 was published very early indeed, at the end of January 2020. Providing a brief description, the novel coronavirus SARS-CoV-2 is equipped with a receptor-binding domain (RBD), a structure similar to the ‘original’ SARS-CoV that emerged for the first time in 2002. Functionally important ORFs (1a and 1b) and other main structural proteins, including the spike (S), membrane (M), envelope (E), and nucleocapsid (N) proteins, are also well interrelated and clearly annotated. According to previous reports, the M and E proteins are essential for virus assembly, while the S protein is crucial for affinity and attachment to host cells, as the RBD of the S protein enables binding with angiotensin-converting enzyme 2 (ACE2). The S protein on the surface of the viral particles and has been reported to be highly immunogenic. The N protein, the main structural protein of SARS-CoV-2 is responsible for the transcription and replication of viral RNA, the packaging of the encapsulated genome into virions, and interactions with the cell cycle of host cells. In addition, coronaviruses contain the N protein, which has a substantial immunogenic ability and is abundantly expressed during viral infection (Figure 1). The S/N proteins are targeted as potential antigens for serodiagnosis of COVID-19, similarly to other diagnostic methods that were implemented for diagnosing SARS disease, based on S/N proteins [7].

## 2. Reference Molecular RT-qPCR Assay for Validated COVID-19 Diagnosis

At present, work is going on worldwide to develop new methods which will simplify and speed up the detection of novel coronavirus. Currently, there are nearly 200 commercially available genetic tests, with further companies awaiting the completion of the procedure and Food and Drug Administration (FDA) or In Vitro Diagnostics (IVD) certificate to be issued. It is worth noting that a large portion of available tests are provided merely with the Research Use Only (RUO) certificate, which in fact does not imply anything concerning test quality or its validation. More rigorous tests apply to products certified as IVD, complying with the ISO13485 norm. In addition, IVD reagents are subject to local regulations, such as CE marking in Europe, and hence they should comply with Directive 98/79/EC of the European Parliament and of the Council of 27 October 1998 on in vitro diagnostic medical devices, with an extensive evaluation, including the validation of clinical samples.

Real Time RT-qPCR genetic tests are meant for the identification and differentiation of SARS-CoV-2 in the material/specimens collected from patients with COVID-19 symptoms, by detecting RNA sequences unique for SARS-CoV-2. The genetic material of the virus is extracted from specimens (sputum, tracheal aspirate or bronchoalveolar lavage, swabs from nasopharynx and pharynx, blood, urine, or stool) and amplified by means of the PCR technique in real time, with the use of reverse transcription (RT), and detected by means of fluorescent reporter probes specific to SARS-CoV-2 [8,9].

At present, RT-qPCR tests are available in the world market, which are meant to detect *ORF1ab*, *E, N*, or *S* gene sequences, in various combinations. Those tests differ in sensitivity, stability, and examination time. The test protocol is complex and costly, being mainly suitable for large, centralized diagnostic laboratories. Tests usually take 4–6 h, yet the logistic requirements concerning sending clinical specimens imply that execution time is 24 h at the most [10].

In accordance with WHO recommendations, RT-qPCR tests must enable the detection of three genes in a single reaction: *E* gene, *N* gene, and *RdRP* gene. This allows us to detect viruses from the beta-coronavirus group (*E* gene), as well as to identify SARS-CoV-2 virus (*N gene* and *RdRP, ORF1ab*). Such a design guarantees double confirmation in cases of infection, it also limits the risk of obtaining false negative results in case of detecting only one target for SARS-CoV-2. This reduces the possibility of obtaining doubtful results, in which case the necessity of verification occurs [11]. A comparison review of RT-qPCR dedicated to SARS-CoV-2 genes target points is presented in Table 1.

The standard protocol with the application of the RT-qPCR method is demanding and time-consuming. For that reason, scientists constantly strive to invent more up to date modifications of the RT-qPCR tests, which would cut down the time required for analyses [13].

Bosch Healthcare Solutions announced that they had developed a quick test, which may provide the results in 2.5 h; it is a fast testing kit for the Vivalytic platform. It is a fully automated PCR test, which is performed by simply inserting the swab into a cartridge, which is subsequently analyzed by the machine. This universal platform for molecular laboratory diagnostics, with the option for various samples testing and different analytical methods, can be carried out as an entirely automated mode within a short time. Unfortunately, the equipment has not been available for many healthcare institutions yet, and the diagnostic cartridges are dedicated solely to the specific analyzing device [14].

BioMaxima has invented a test whose main advantages include a short waiting time; the results can be obtained in a mere 2 h. Moreover, the analytical sensitivity of the test, being at the level of ≥10 RNA copies per reaction, is higher than in other comparable systems. It seems important that the molecular test kit contains reagents with substantially enhanced stability, which allows us to transport it safely and store it at room temperature, whereas many other tests available on the market need to be stored in a freezer and to be transported in dry ice. Storing such reagents, even for a short time, in conditions that deviate from those indicated is risky and may result in generating false negative test results [15].

The latest technology of molecular tests, developed by scientists from Oxford University, is more sensitive than the previous ones and it implies the possibility of examining patients at earlier stages, reacting more quickly, and effectively preventing the spread of coronavirus. This new test for diagnosing SARS-CoV-2 provides rapid results within 30 min, whereas the fastest current methods that concentrate on viral RNA give results in 1.5 to 2 h, i.e., three times faster than the presently quickest testing methods, and which requires the application of relatively simple technical devices. Apart from those advantages, the scientists responsible for the test’s development claim that it may even help detect patients infected with coronavirus at earlier stages, in comparison with current methods, while its results can be read with the “naked eye”, which makes it more available for a wider spectrum of healthcare units and specialists. This fast-molecular test has been recently registered by the FDA and utilizes a diagnostic system, which, in recent years, proved to function perfectly in diagnosing multiple infections (including Hepatitis C Virus—HCV and influenza) [16]. RNA detection may be performed by means of RT-qPCR or reverse transcription–loop amplification (RT–LAMP). A standard RT-qPCR test takes 90 to 120 min on average to test a set of samples, whereas LAMP may be completed in 30 min. Indirect isothermal amplification (LAMP) is a fast technology of DNA amplification, which is applied in the detection of pathogens such as viruses or bacteria. LAMP reaction usually takes place at constant temperature, and the target DNA may be amplified in 30 min. LAMP method utilizes four or six starters for binding six regions of target DNA, its specificity is exceptionally high. Because SARS-CoV-2 is a RNA-type virus with a length of some 30 kb, a single reverse transcriptase (RT) reaction and LAMP may, jointly, significantly shorten the reaction time, omitting the stage of purifying cDNA from reverse transcriptase, and thanks to this, SARS-CoV-2 may be quickly detected. It has to be noted that similarly, a one-step RT-qPCR method does not require cDNA purification (a single stage reaction). The team from Oxford University developed four sets of LAMP starters (comprising six starters in each set/kit) focused on viral RNA of SARS-CoV-2 in the regions of ORF1ab, S gene, and N gene. For the interpretation of results, a colorimetric method has been applied, which enables the reading of the results of viral RNA amplification by the naked eye, without the necessity of using expensive equipment. Moreover, the sensitivity of the method may be 80 copies of viral RNA per 1 mL in the sample and single stage process (without separate RNA extraction), which enables the amplification of RNA directly from the sample [17,18].

Performing examinations with the use of fast molecular tests may be particularly useful in Emergency Departments and admission rooms. POCTs are needed to speed up the process of taking clinical decisions and decrease the workload for centralized testing laboratories [16]. POCT means that test results are instantly delivered in the patient care settings, such as hospitals, urgent care centers and emergency rooms, instead of the use of expensive and time-consuming laboratory processes.

Additionally, the American company Cepheid developed a quick test which, as they declare, takes 45 min, while the Dutch pharmacists owning Qiagen developed a 1-h test. The US Food and Drug Administration has issued an emergency authorization of use for Cepheid’s point-of-care COVID-19 diagnostics, Xpert Xpress SARS-CoV-2, according to a statement from the agency. The test was designed to provide detection of novel coronavirus within a short period of 45 min, using specimens from a nasopharyngeal swab, nasal wash/aspirate. Another POCT, the Xpert Xpress SARS-CoV-2 test cartridge is dedicated to detecting SARS-CoV-2 nucleic acid utilizing RT-qPCR and does not require the use of reagents. In the contrary, the GeneXpert System needs the test to be run in a CLIA-approved laboratory (Clinical Laboratory Improvement Amendments) or in a selection of patient care settings [19].

It is equally important to understand that RT-qPCR tests have also certain limitations. They are the most useful in case of positive results, although they appear to have less diagnostic value in situations when COVID-19 must be ruled out. Negative RT-qPCR test results do not necessarily indicate that person has not contracted an infectious disease, as other individual factors also need to be considered, such as exposure risk and potential laboratory errors. The false negative results may occur if the sample has not been properly collected, transported, or handled/treated, or as a result of the improper extraction of nucleic acid from clinical materials [9]. Moreover, the false positive results can be related to the situation where the sample contains inhibitors of amplification, or an insufficient amount of virus molecules. On the contrary, a false positive result may be due to the cross-contamination of the sample during its handling or preparation, or between patient samples. What is more, the influence of vaccines, antiviral drugs, antibiotics, chemotherapeutic agents, or immunosuppressive drugs has not been taken into consideration. Moreover, the set/kit does not exclude diseases caused by other bacterial or viral pathogens. The negative results do not exclude infection and should not be the sole basis for patients’ treatment. Test results when using the kit serve clinical purposes only. Clinical diagnosis and treatment of patients should be considered in conjunction with the manifestations and symptoms, medical history, response to treatment or other laboratory tests [9]. In general, the sensitivity of this molecular method may vary depending on specimen collection (broncho-alveolar lavage, sputum, nasal swabs or throat swabs) and, what is more, the accuracy depends upon stage of infection, the rate of SARS-CoV-2 multiplication, and also the degree of clearance. Interestingly, the provided values of RT-qPCR accuracies seem to be higher for in vitro validation by utilizing SARS-CoV-2-dedicated primers and cultures in highly controlled laboratory settings [20].

It is assumed that the optimal outcome and ‘clear-cut diagnostic gold standard’ might depend upon the development of hybrid assays in order to minimize the fraction of false negative results. The enhanced RT-qPCR test combined with serological immunoassays (either antibody-detecting or antigen-detecting), would bring an additional diagnostic value for accurate and quick COVID-19 diagnosis in vitro diagnostics, when the human body reacts to an infectious bio-stimulant [21].

### Evaluation of Molecular RT-qPCR Tests that Detect the RNA Nucleic Acid of SARS-CoV-2

The progress made in recent years in the diagnostics of infections enables the application, often simultaneously, of numerous methods in order to detect respiratory viruses effectively. The choice of suitable tests depends upon the type of virus to be detected, the presumed number of antigens, patient population to be tested, as well as the technical abilities and experience of the testing unit. The validation of RT-qPCR tests seems to be an important step in combating the new coronavirus. One of the most significant parameters regarding the efficiency of the diagnostic procedure is most probably the one related to the minimum amount of analyte, which may be detected and quantitatively determine by means of a particular test. The parameters describing those properties are known as limit of detection (”LoD”) and limit of quantification (”LoQ”). In many laboratories, LoD is used interchangeably with “sensitivity”, “analytical sensitivity”, or/and “detection limit.”. This may be misleading, though, as “sensitivity” is also understood and used in other ways. Sensitivity and clinical (diagnostic) specificity are two different parameters. Diagnostic sensitivity is the proportion of true positive results to the sum of true positive and true negative results. A sensitivity ratio of 100% implies that all the sick people have been identified. The specificity of a test is the proportion of true negative results to the sum of true negative and false positive ones. The specificity of 100% implies that all healthy people in a given test have been correctly identified as healthy/not having the condition. As indicated in Table 2, sensitivity and specificity of tests is already about 100%, with a few exceptions, still one can notice differences in analytical sensitivity of the test (LOD). The vast majority of molecular tests kits contain a so-called ‘internal positive control’ (IPC), that can be utilized either as an extraction control or internal control. The IPC is essential for an evaluation of whether the genetical material extraction procedure and amplification stage were carried out correctly. The failure in IPC detection using a patient’s specimen may indicate the improper extraction of nucleic acid from clinical material as a consequence of nucleic acid loss or the transposition of PCR inhibitors and the lack of sufficient biological material in the collected sample. It needs to be noted that many molecular tests may not provide sets for extraction stages, and they need to be acquired separately. However, all tests reviewed here that detect the RNA of SARS-CoV-2 contain IPC.

Another important factor which describes a diagnostic test is the analytical specificity. It is the ability of a test to detect a specific target, e.g., a virus. It is of great importance to check whether the starters used in PCR test are specific to a given virus. There are two components of analytical sensitivity: cross-reactivity and interference. Cross-reactivity may occur if, in a sample collected from a patient, there are organisms which are genetically related that imitate the virus analysed, which causes test starters to cross react. As shown in Table 3, the producers have analysed their tests from the perspective of cross-reactivity; unfortunately, that analysis is selective and does not concern the same micro-organisms.

## 3. Emerged Rapid Immunodiagnostic (Serology Immunoassays) Tests

Immunological methods are, most often, the chemiluminescent assaying of immunoglobulin IgG and IgM for SARS-CoV-2 from blood on an analyzer, or immunochromatographic assessment in the form of rapid POCTs, not requiring additional equipment. The methods of detecting anti-SARS-CoV-2 antibodies, despite the ongoing research to develop them further, may or even should be applied during the coronavirus epidemics [22,23]. After about a week from the first clinical manifestations, the sensitivity of molecular diagnostics (PCR) diminishes gradually for SARS-CoV-2 infections, due to the decreasing amount of virus particles in the respiratory tract epithelium. In such cases, patients may have false negative results, despite the ongoing infection.

### 3.1. Lateral Flow Immunoassay

Among the many contemporary technologies available, special attention should be paid to rapid lateral flow immunoassay (LFIA), also referred to as immunochromatographic tests. Perhaps they are not so much appreciated in the scientific community as PCR methods or Enzyme-Linked Immunosorbent Assay (ELISA tests), despite the fact that they do find application in diagnostics more and more often. They differ, depending on the type of test, but the basic principle of their action is invariably the same—they make use of the unique property that antibodies possess, that of selective binding to a specific particle or group of similar particles (antigen). LFIAs provide an uncomplicated and relatively inexpensive tool meant for detecting the presence (or absence) of a given component in the examined specimen, such as the presence of a virus in an analyzed blood sample. Examination with the use of those tests is possible for various types of test material—whole human blood, blood plasma, serum, stool, urine, sweat, cerebrospinal fluid, or even tears [9]. The test principle is based on an immunological method, using specific antibodies, most often in complex with colloidal gold, where a drop of the examined substance first moves along the nitrocellulose membrane using capillary phenomena. After the sample is absorbed by the membrane, the antigen (should the test prove to be positive) binds to the colloidal gold complex and respective antibodies. Consequently, the effect of that reaction is the formation of a complex, which will be detected by the test. The interpretation of results consists of confirmation or ruling out of the presence of antigens in the examined sample, based on color test strips that appear in the test [21,22]. The brief comparison of advantages and disadvantages of immunochromatographic tests is presented in Table 4.

At present, most immunoenzymatic tests available are based on the immunochromatic technique. The difference between those tests depends upon the molecule assayed (p/c or antigens), structure, performance time, and diagnostic material. It may be supposed that, in future, rapid tests will enable easier and quicker diagnostics of many diseases, without the necessity of performing tedious and complicated procedures. This would reduce the waiting time for obtaining results, and accelerate decision making regarding suitable treatment [27,28,29].

### 3.2. Immunoenzymatic and Immunofluorimetric Assays

The detection of specific SARS-CoV-2 serum antibodies allows for a rapid, cost-effective, and reasonably sensitive clinical diagnosis of COVID-19, as immunoglobulins such as IgM provide the initial humoral response during the first stage of viral infection, prior to the onset of the adaptive, high-affinity IgG response essential for long-term immunological memory. Research indicates that after SARS infection, antibodies of the IgM class may be detected in patient’s blood about 6 days after the infection, while IgG may be already detected after 8 days. As SARS-CoV-2 belongs to the same large family of viruses, which includes those causing Middle East Respiratory Syndrome (MERS) and Severe Acute Respiratory Syndrome (SARS), it should be assumed that the process of producing antibodies will be similar to that in case of other viruses belonging to that family, while the detection of IgG antibodies and IgM antibodies acting against SARS-CoV-2 may be an indication of infection. Moreover, the detection of IgM antibodies usually indicates a recent exposure to SARS-CoV-2, whereas the detection of IgG antibodies in case of COVID-19 indicates exposure to the virus some time ago [30].

Serological tests, detecting solely the IgM class of antibodies, should find applications for diagnostic purposes. When using tests which detect both IgM and IgG antibodies, one should remember that a positive result may be the evidence of past infection, not active infection (Table 5). Negative results from serological tests do not exclude SARS-CoV-2 infection, as the ‘window period’ (delay in the production of antibodies) may exceed 7 days. Serological tests may also give false positive results. This may be the case of past or ongoing infection with virus strains other than SARS-CoV-2, such as coronavirus HKU1, NL63, OC43, or 229E. Moreover, the first tests assessing the titer of IgA class antibodies have been launched, which, from the perspective of immunology, is of extreme importance, as it provides the possibility of testing for antibodies in a material other than blood samples collected from patients, e.g., respiratory tract secretions.

Serological tests are thus applied as an adjunctive method, for monitoring the epidemiological situation, yet they may be performed faster and are less costly than genetic tests. This diagnostic method has a limited sensitivity, yet efforts to improve it are ongoing, as it is useful for monitoring the infection. Due to insufficient data concerning, among others, the dynamics of immunological response to infection and the diagnostic value of available tests for detecting IgM and IgG class antibodies (comprising sensitivity, specificity, positive and negative predictive value), in many countries, it is currently not recommended to use serological tests for diagnostic purposes. Table 6 displays the evaluation of serological tests validated by the FDA, and Table 7 provides a comparison of the selected immunochromatographic tests for SARS-CoV-2 based on sensitivity, specificity, sample type and test performance time.

In serological IVD, the so-called ‘window period’ is of much importance—that is, the time when specific antibodies are not yet detectable in a patient’s blood. In the initial phase of an infection, the production of antibodies is initiated, but their level is still low, and thus test results may prove negative. Anti-SARS-CoV-2 antibodies appear quite late, i.e., a few days after clinical manifestations, which is why serological tests cannot be used as a basic tool in diagnosing SARS-CoV-2 infections [32].

The detection of specific anti-SARS-CoV-2 antibodies is possible about 10 days after the first clinical manifestations of infection. Thus, this serves the purpose of confirming contact with coronavirus, and is of enormous importance from an epidemiological perspective, as it allows us to detect subjects who had the infection but were asymptomatic or oligosymptomatic, and could have been the source of infection for other people (“silent” carriers/vectors). It also allows us to determine the spread of COVID-19 infection in the population. A comparative review of currently available serological immunodiagnostic COVID-19 tests is presented in Table 8.

### 3.3. Protein Microarray Method (PMM)

The protein microarray method (PMM) is a proteomic screening technique serving the purpose of simultaneous/parallel quantitative and qualitative analysis of mixtures containing many proteins. The chip used is composed of a supporting surface (e.g., modified glass plate, nitrocellulose membrane, or microtitration plate), on which the matrix of ‘capturing’ proteins is immobilized in accordance with the determined formula, performing the function of a probe. The material captured may be antibodies, enzymes, or ligands. Protein analyte, added to the matrix, may be modified by means of various types of markers (among others: radioisotopes, fluorescence, luminescence markers). The interaction between micromatrix proteins and analyte induces an analytical signal, registered by means of suitable equipment. The advantages of protein micromatrices include the short time required for analysis, modest consumption of samples and reagents, high sensitivity, automation of testing. They allow us to investigate the functions and effects of proteins on large scale [33,34].

The newly developed PEPperCHIP^®^ SARS-CoV-2 Proteome Microarray (PEPperPRINT) based on the SARS-CoV-2 genome derived from the virus isolate Wuhan-Hu-1 (GenBank ID: MN908947.3) enables us to serologically screen about 5000 individual peptides spanning the whole viral proteome. Sequences of ORF1ab protein, surface glycoprotein, ORF3a protein, coat protein, membrane glycoprotein, ORF6 protein, ORF7a protein, ORF8 protein, nucleocapsid phosphoprotein, and ORF10 protein have been elongated and connected by means of neutral GSGSGSG connectors. The elongated protein sequences have been transcribed into 4883 different peptides composed of 15 amino acids printed in two repetitions (9766 peptide sites) in order to obtain high-resolution epitope data. In the case of SARS-CoV, which emerged in 2002, it was demonstrated that the application of PMM is characterized by enhanced sensitivity, as more Chinese subjects were diagnosed as SARS-positive when applying PMM in comparison with ELISA tests. The PMM seems to be more reliable, as it allows us to determine more protein antigens of the virus [35]. An additional advantage of such tests is the possibility of automating the entire analytical process. Unfortunately, the method has several imperfections, including the repeatability of the test itself, while the cost of the testing and equipment used for micromatrix diagnostics is significant, which makes the PMM method less accessible in standard laboratory settings [36].

Undoubtedly, the scalable and reliable POCTs used in the community and outside laboratories would have the opportunity to reduce the time to obtain COVID-19 diagnostic results and might support early implementation of isolation resources and infection control measures. From a medical workforce point of view, antibody- and antigen-detecting tests appear to have a significant role in identifying healthcare workers who recovered from SARS-CoV-2 infection, to assess their suitability to provide frontline health services. On the other hand, the false negative serology tests could cause spread of COVID-19, unjustified reassurance and a change in public behavior. Undoubtedly, the negative results of serology tests (swabs) should not be treated as definitive for ‘ruling out’, but a positive test results are beneficial to ‘rule in’ coronavirus infection. The sufficient accuracy of serology tests used more widely will allow us to make an appropriate decision regarding lifting restrictions on a national level. Nevertheless, the wider implementation of POCD will ultimately support public health strategies about the potential lifting of social distancing restrictions in future [30]. Moreover, with the limited data available at present, for antibody-detecting and antigen-detecting rapid POCD, further intense research into their potential diagnostic utility is highly encouraged. If these tests demonstrate adequate accuracy, it is likely that they could potentially be used as triage tests for quick identification of patients who are suspected of contracting COVID-19, reducing the need for expensive reference molecular PCR testing [4,6]. Finally, it is worth noting the psychological and behavioral consequences of correctly established and known immunity status within the community.

## 4. The Currently Developing Concepts of COVID-19 Diagnostics

### 4.1. Clustered Regularly Interspaced Short Palindromic Repeats (CRISPR/Cas13) Technology

Recently, a test has been developed based on Clustered Regularly Interspaced Short Palindromic Repeats (CRISPR/Cas12a), with visual readouts. The test can detect 10 copies of the virus gene in merely 45 min, providing simple and reliable diagnostic method, which additionally demonstrates high sensitivity and specificity. In order to enable fast diagnosis at the hospital or on admission, the test has been provided with an ssDNA reporter labeled with a blank green fluorescent particle, which will be split off by the Cas12a protein, when the nucleic acid of SARS-CoV-2 is found in the detection system. In this way, a green signal of fluorescence will be obtained, visible with the naked eye, in the light, with a wavelength of 485 nm. The high specificity of the test was obtained by designing 15 crRNAs, which may distinguish the polymorphisms of a single nucleotide with other viruses related to SARS in four domains: ORF1a or ORF1b, N, and E genes [37].

### 4.2. Exhaled Breath Condensate (EBC)

The search for new diagnostic methods is continued. Because SARS-CoV-2 is spread by droplets, while the diagnostic sensitivity and specificity index for RT-qPCR tests is the highest for broncho-alveolar lavage (BAL) (higher than for sputum or nose/throat swabs), scientists recently proposed the use of exhaled breath condensate (EBC) for diagnostic purposes. EBC is most similar in its biochemical composition and origin to BAL. Examinations with the use of EBC are a relatively novel diagnostic method, used mainly to assess inflammation within the respiratory tract, and its components reflect the composition of the fluid lining the bronchi and alveoli. EBC is a condensed form of small droplets of the fluid lining the lungs, which is usually exhaled, it contains various components, from small ions to proteins and organelles; it may even contain viruses, fungi, and bacteria. Taking samples of the condensate is safe, totally non-invasive, easy to perform, it may be repeated many times, it may also be performed on patients with severe complications of COVID-19; its enormous advantage is that in may be performed in small children, with the use of special facial masks. The course of the examination is such that the patient is requested to exhale, when breathing uneventfully for some 10-15 min, to an apparatus provided with a cooling system, which allows us to accumulate the condensate in which SARS-CoV-2 may be detected. The application of this method seems justified from a public health perspective. The false positive results that are presently obtained contribute the continued spread of the virus worldwide [38,39].

## 5. Conclusions

The optimization of laboratory diagnostics is the most dynamically developing field in the time of the COVID-19 pandemic, supporting contemporary medicine, government decisions and healthcare strategies. The efforts of scientist–clinician teams focus, first of all, upon implementing the most reliable diagnostic tools; however, because COVID-19 is a new nosological entity, there are not enough data as of yet that would enable the determination of standards for the interpretation of serological POCTs. As with any other infectious diseases, the diagnostic value of a test is not only about the method of collecting the material, the quality of the sample and the equipment applied. Equally essential pre-analytical considerations are also the time point when a sample is collected, as well as a suitable procedure (storage and handling) prior to analysis, from the moment of collecting the biological material to the assaying stage.

## Figures and Tables

**Table 1:** Comparison of RT-qPCR dedicated for SARS-CoV-2 genes target points [12] (updated 13 June 2020, online sources).

Developer	Name of the Kit	Gene						Regulatory
		*ORF1a*	*ORF1ab*	*RdRP*	*E*	*N*	*S*	
**Manual test**								
1drop Inc.	1copy™ COVID-19 qPCR Kit							CE-IVD
AB ANALITICA srl	REALQUALITY RQ-2019-nCoV							CE-IVD
ADT Biotech	LyteStar 2019-nCoV RT-PCR Kit 1.0							RUO
altona Diagnostics	RealStar^®^ SARS-CoV-2 RT-PCR Kit							USA EUA; CE-IVD
Atila Biosystems Inc.	Atila iAMP^®^ COVID Detection Kit							USA EUA
BIOMAXIMA S.A.	SARS-CoV-2 Real Time PCR LAB-KIT™							CE-IVD
bioMérieux	SARS-COV-2 R-GENE^®^							USA EUA; CE-IVD; RUO
Bioneer	AccuPower^®^ SARS-CoV-2 Real-Time RT-PCR							CE-IVD
BGI Health (HK)	Real-time fluorescent RT-PCR kit 2019-nCoV							USA EUA; CE-IVD; Canada
CerTest Biotec, S.L	VIASURE SARS-CoV-2 Real Time PCR Kit							CE-IVD
CerTest Biotec, S.L	VIASURE SARS-CoV-2 S gene Real Time PCR Kit							CE-IVD
Co-diagnostics	Logix Smart Coronavirus disease 2019							CE-IVD
CTK Biotech, Inc.	Aridia COVID-19 Real Time PCR Test							CE-IVD
DAAN Gene Co	Detection Kit for 2019 Novel Coronavirus							CE-IVD; China
Edinburgh Genetics	COVID-19 Real-time PCR Testing Kit							CE-IVD; China FDA
Gencurix Inc.	GenePro COVID-19 Detection Test							CE-IVD
Genomictree, Inc.	AccuraTect RT-qPCR SARS-CoV-2							CE-IVD
KH Medical	RADI COVID-19 Detection Kit							CE-IVD
KRISHGEN	SARS-CoV-2 (COVID-19) Real-Time PCR Kit							RUO
Liming Bio-Products	SrongStep^®^ Novel Coronavirus (SARS-CoV-2)							CE-IVD
PerkinElmer Inc.	PerkinElmer^®^ SARS-CoV-2 Realtime RT-PCR							CE-IVD; WHO-EUL
Primerdesign Ltd.	COVID-19 genesig Real-Time PCR assay							CE-IVD; USA EUA;WHO EUL
R-Biopharm AG	RIDA^®^ GENE SARS-CoV-2 RUO (PG6815RUO)							RUO
SD BIOSENSOR Inc.	STANDARD M nCoV Real-Time Detection Kit							CE-IVD; USA EUA; Brazil
TIB/Roche Diagn.	LightMix Modular SARS-CoV-2 (COVID19)							RUO
TIB/Roche Diagn	LightMix Modular SARS-CoV-2 (COVID19)							RUO
TIB/Roche Diagn	LightMix Modular SARS-CoV-2 (COVID19)							RUO
SD BIOSENSOR	STANDARD M nCoV Real-Time Detection kit							USA EUA; CE-IVD; Brazil
Seegene, Inc.	Allplex 2019-nCoV assay							USA EUA; CE-IVD; Canada
Sansure Biotech Inc	Novel Coronavirus (2019-nCoV) Nucleic Acid							USA EUA; CE-IVD; China
Sente Biolab Sentelig	COVID-19 qRT PCR Detection Kit							CE-IVD
Shanghai ZJ Bio-Tech	Liferiver Novel Coronavirus Multiplex RT-PCR							CE-IVD, China FDA
Thermo Fisher	TaqPath™ COVID-19 CE-IVD RT-PCR Kit							CE-IVD
**Automated Lab-based, near-POC NAT or POC NAT**								
3D Biomedicine	DMed 2019-nCoV RT-qPCR Detection Kit							US FDA—CE-IVD
Abbott Molecular Inc.	Abbott RealTime SARS-CoV-2 EUA test							US FDA—CE-IVD
Cepheid	Xpert Xpress SARS-CoV-2							US FDA-EUA
Roche Molecular Dia	cobas^®^ SARS-CoV-2							US FDA—WHO EUL
Sente Biolab	Senteligo COVID-19 qRT PCR Detection Kit							CE-IVD
Solgent Co.Ltd	DiaPlexQ™ Novel Coronavirus (2019-nCoV)							CE-IVD
Star Array Ptd. Ltd.	8-min RT-qPCR direct PCR testing							RUO
Veredus Laboratories	VereCoV™ Detection Kit and VerePLEX™							CE-IVD

**Table 2:** The table shows results of independent evaluation to verify the clinical performance of tests. The research was carried out at the University Hospitals of Geneva [12] (updated 13 June 2020, online sources).

Developer	Name of the Kit	Gene	Clinical Sensitivity	Clinical Specificity	Limit of Detection LOD (Copies/Reaction)
**altona Diagnostics**	RealStar^®^ SARS-CoV-2 RT-PCR Kit 1.0	*E*	92%	100%	1–10
		*S*	92%	100%	1–10
**Atila BioSystems Inc.**	Atila iAMP COVID-19 Detection (isothermal detection)	*ORF1ab*	100%	99%	20–100
		*N*	100%	100%	1–10
**BGI Health (HK) Co. Ltd.**	Real-time Fluorescent RT-PCR kit for detection 2019-nCOV (CE-IVD)	*ORF1*	100%	99%	1–10
**bioMérieux**	ARGENE^®^ SARS-COV-2 R-GENE^®^	*N*	100%	100%	10–50
		*RdRP*	96%	100%	10–50
**BIONEER**	AccuPower^®^ SARS-CoV-2 Real-Time RT-PCR Kit	*E*	100%	100%	10–50
		*RdRP*	100%	100%	10–50
**Boditech Med. Inc.**	ExAmplar COVID-19 real-time PCR kit (L)	*E*	100%	100%	10–50
		*RdRP*	90%	100%	50–100
**CerTest Biotec**	VIASURE SARS-CoV-2 Real Time PCR Detection Kit	*ORF1ab*	98%	100%	10–50
		*N*	100%	100%	1–10
**DAAN Gene Co. Ltd.**	Detection Kit for 2019 Novel Coronavirus (2019-nCoV) RNA (PCR-Fluorescence Probing)	*ORF1*	100%	96%	1–10
		*N*	100%	98%	1–10
**EUROIMMUN**	EURORealTime SARS-CoV-2	*ORF1ab/N*	100%	98%	1–10
**GeneFirst Ltd.**	The Novel Coronavirus (2019-nCoV) Nucleic Acid Test Kit	*ORF1*	100%	99%	1–10
		*N*	98%	100%	1–10
**KH Medical Co. Ltd.**	RADI COVID-19 Detection Kit	*S*	100%	100%	1–10
		*RdRP*	100%	100%	10–50
**Primerdesign Ltd.**	Coronavirus COVID-19 genesig^®^ Real-Time PCR assay	*RdRP*	100%	100%	1–10
**R-Biopharm AG**	RIDA^®^ GENE SARS-CoV-2 RUO	*E*	100%	100%	1–10
**SD Biosensor Inc.**	STANDARD M nCoV Real-Time Detection Kit	*E*	100%	97%	1–10
		*ORF1*	100%	99%	1–10
**Seegene Inc.**	Allplex™ 2019-nCoV Assay	*E*	100%	100%	1–10
		*N*	100%	100%	1–10
		*RdRP*	100%	100%	1–10
**Shanghai Kehua Bio-Engineering**	KHB Diagnostic kit for SARS-CoV-2 Nucleic Acid (Real-time PCR)	*ORF1*	100%	100%	1–10
		*N*	100%	100%	1–10
		*E*	100%	100%	1–10
**Tib Molbiol**	ModularDx Kit SARS-CoV (COVID19) E-gene (Tib Molbiol) + LightCycler Multiplex RNA Virus Master (Roche)	*E*	100%	100%	1–10
**Vela Diagnostics**	ViroKey™ SARS-CoV-2 RT-PCR Test	*RdRP*	94%	100%	10–50
		*ORF1*	100%	100%	1–10

**Table 3:** The analytical specificity of the test with respect to cross reactivity with other pathogens than Severe Acute Respiratory Syndrome Coronavirus 2 (SARS-CoV-2) (updated 13 June 2020, online sources according to manufacturers’ specification).

Microorganism	Name of Test												
	1drop Inc. 1copy™ COVID-19 qPCR Kit	Altona Diagnostics RealStar^®^ SARS-CoV-2 RT-PCR Kit	Atila iAMP^®^ COVID Detection Kit	CerTest Biotec, S.L VIASURE SARS-CoV-2	Co-diagnostics Logix Smart Coronavirus Disease 2019	Edinburgh Genetics COVID-19 Real-time PCR Testing Kit	Liming Bio-Products SrongStep^®^ Novel Coronavirus (SARS-CoV-2)	PerkinElmer^®^ SARS-CoV-2 Realtime RT-PCR	SD BIOSENSOR Inc. STANDARD M nCoV Real-Time Detection Kit	Seegene, Inc. Allplex 2019-nCoV Assay	Thermo Fisher Scientific TaqPath COVID-19 Combo Kit	Abbott RealTime SARS-CoV-2 EUA test	Cepheid Xpert Xpress SARS-CoV-2
Human coronavirus NL 63	-	-	-	-	-	-	-		-	-	-	-	-
Human coronavirus OC229E	nd	-	-	-	-	-	-	-	-	-	-	-	-
Human coronavirus OC43	-	-	-	-	-	-	nd	-	-	-	-	-	-
Human coronavirus HKU1	-	nd	-	-	-	-	-	nd	-	-	-	-	-
Human Metapneumovirus (hMPV)	-	-	-	-	-	nd	nd	nd	nd	-	-	-	-
SARS-coronavirus	-	-	-	-	-	nd	nd	-	-	-	-	-	-
MERS-coronavirus	-	-	-	-	-	nd	nd	-	-	-	-	-	-
Parainfluenza virus 1	-	-	-	-	-	nd	nd	-	-	-	-	-	-
Parainfluenza virus 2	-	-	-	-	-	nd	nd	-	-	-	-	-	-
Parainfluenza virus 3	-	-	-	-	-	nd	nd	-	-	-	-	-	-
Parainfluenza virus 4	-	-	-	nd	-	nd	nd	-	-	-	-	-	-
Influenza A virus	-	-	nd	-	-	-	-	-	-	-	-	-	-
Influenza B Virus	nd	-	-	-	-	-	-	-	-	-	-	-	-
Adenovirus	nd	-	nd	-	-	-	nd	-	-	-	-	-	-
Enterovirus (e.g., EV68)	-	-	-	nd	-	nd	nd	-	-	-	-	-	-
Respiratory syncytial virus A	nd	-	-	-	-	-	-	-	-	-	-	-	-
Respiratory syncytial virus B	nd	-	-	-	-	-	-	-	-	-	-	-	-
Rhinovirus	-	-	-	-	-	nd	nd	-	-	-	-	-	-
Chlamydia pneumoniae	-	-	-	-	-	nd	nd	-	-	-	-	-	-
Hemophilus influenzae	-	-	nd	-	-	nd	nd	-	-	-	-	-	-
Legionella pneumophila	-	-	nd	-	-	nd	nd	nd	-	-	-	-	-
Mycobacterium tuberculosis	-	nd	nd	-	-	nd	nd	nd	-	-	-	-	-
Streptococcus pneumoniae	-	-	nd	-	-	nd	-	nd	-	-	-	-	-
Streptococcus pyogenes	-	-	nd	nd	-	nd	nd	-	-	-	-	-	-
Bordetella parapertussis	nd	nd	nd	-	nd	nd	nd	nd	nd	nd	nd	nd	nd
Bordetella bronchiseptica	nd	nd	nd	-	nd	nd	nd	nd	nd	nd	nd	nd	nd
Bordetella pertussis	-	-	-	-	-	nd	nd	nd	-	-	-	-	-
Mycoplasma pneumoniae	-	-	nd	-	-	-	nd	-	-	-	-	-	-
Pneumocystis jirovecii (PJP)	-	-	nd	-	-	nd	nd	nd	-	-	-	-	-
Candida albicans	-	-	nd	nd	-	nd	nd	nd	nd	-	-	-	-
Pseudomonas aeruginosa	-	-	-	nd	-	nd	nd	nd	-	-	-	-	-
Staphylococcus epidermis	-	nd	nd	nd	-	nd	nd	nd	-	-	-	-	-
Staphylococcus salivarius	-	nd	-	nd	-	nd	nd	nd	-	-	-	-	-
Staphylococcus aureus	nd	nd	nd	-	-	nd	nd	-	nd	nd	-	nd	-
Human immunodeficiency virus type 1,2	nd	nd	nd	nd	nd	nd	nd	-	nd	nd	nd	nd	nd
Hepatitis virus (A, B, C)	nd	nd	nd	nd	nd	nd	nd	-	nd	nd	nd	nd	nd

No data available (nd); does not cross-react with analyzed pathogen (-).

**Table 4:** The brief comparison of advantages and disadvantages of immunochromatographic tests [24,25,26,27].

Advantages	Disadvantages
Short reaction time for most tests, amounting to 5–20 min	Suboptimal sensitivity, results often false negative, particularly during enhanced activity of the virus
Simple and comfortable to use and perform. Some tests may be performed in outpatient clinics or at patient’s bed.	Despite substantial specificity sometimes the results are false negative, particularly when the virus is not much active.
Reading most often possible with ‘naked eye’.	It is necessary to verify positive or doubtful results.
Small amount of material to be collected, variety of material.	Increased risk of operator becoming infected
“best before” date distant (usually 18 months from manufacturing date)	

**Table 5:** Clinical significance of an IgM/IgG serological test result.

Phase of Infection	Type of Test		
	PCR	IgM	IgG
The window period for a test designed to detect a specific disease	P(+)	N(−)	N(−)
Early stage of infection	P(+)	P(+)	N(−)
Active phase of infection	P(+)	P(+)	P(+)
Late or recurrent stage of infection	P(+)	N(−)	P(+)
Early stage of infection. PCR result may be false negative *	N(−)	P(+)	N(−)
Past infection (recover) *	N(−)	N(−)	P(+)
The recovery stage of infection, or PCR result may be false negative *	N(−)	P(+)	P(+)
No infection and no special symptoms	N(−)	N(−)	N(−)

P(+)—positive; N(−)—negative. * Human coronaviruses (HCoV) OC43, 229E, NL63, and HKU1 may cause false positive ELISA results.

**Table 6:** Evaluations of Coronavirus Disease 2019 (COVID-19) serological tests, including sensitivity, specificity, and predictive value [31] (updated 13 June 2020, online sources according to manufacturers’ specification).

Test Name	Euroimmun SARS-COV-2 ELISA (IgG)	Healgen COVID-19 IgG/IgM Rapid Test Cassette	Biomedomics COVID-19 IgM-IgG Rapid Test kit	Phamatech COVID19 RAPID TEST	Tianjin Beroni Biotechnology SARS-COV-2 IgG/IgM Antibody Detection Kit
Clinical Sensitivity IgM		100%	86.7%	26.7%	83.3%
Clinical Specificity IgM		100%	97.1%	97.5%	100%
Clinical Sensitivity IgG	90%	96.7%	73.3%	86.7%	30%
Clinical Specificity IgG	100%	97.5%	100%	96.2%	100%
Clinical Sensitivity IgM+IgG		100%	96.7%	86.7%	90%
Clinical Specificity IgM+IgG		97.5%	97.1%	93.8%	100%
Positive Predictive Value at prevalence = 5% (IgM+IgG or IgG)	100%	67.8%	63.7%	42.4%	100%
Negative Predictive Value at prevalence = 5% (IgM+IgG or IgG)	99.5%	100%	99.8%	99.3%	99.5%
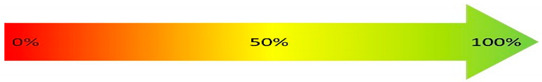					

**Table 7:** Comparison of selected immunochromatographic tests for SARS-CoV-2 based on sensitivity, specificity, sample type and test performance time (updated 13 June 2020, online sources according to manufacturers’ specification).

Developer	Test	Sensitivity:	Specificity:	Sample Size	Time (min)
AccuBioTech Co. Ltd.	Accu-Tell COVID-19 IgG/IgM Rapid Test Cassette	IgG 97.4% IgM 86.8%	IgG 99.3% IgM 98.6%	10 μL of whole blood, serum or plasma	10
AllTest Biotech Hangzhou	2019-nCoV IgG/IgM Rapid Test Cassette	IgG 100% IgM 85%	IgG 98% IgM 96%	10 μL of serum or plasma 20 μL of fingertip blood or whole blood	10
Aytu Bioscience	COVID-19 IgG/IgM Rapid Tes	lgM 89.2% lgG 91.9%	IgM 100% IgG 100%	5 μL of serum or plasma 10 μL of whole blood	2–10
BIOMAXIMA S.A.	2019-nCoV IgG/IgM Rapid Test Cassette	IgG100% IgM 85%	IgG 98% IgM 96%	10–20 μL whole blood, serum or plasma	10–15
BioMedomics, Inc	COVID-19 IgM-IgG Dual Antibody Rapid Test	89%	91%	10–20 μL whole blood, serum or plasma	10–15
Cellex Inc.	Cellex qSARS-CoV-2 IgG/IgM Cassette Rapid Test	93.8%	96.0%	10 μL whole blood, serum or plasma	15–20
Changsha Sinocare Inc.	SARS-CoV-2 Antibody Test Strip (Colloidal Gold Method)	96.3% Serum/Plasma 95.0% Whole blood	99.6% Serum/Plasma 99.2% Whole blood	10 μL of whole blood, serum or plasma	15–20
CTK Biotech	OnSite COVID-19 IgG/IgM Rapid Tes	96.9%	99.4%	10–15 μL of serum or plasma	10–15
Edinburgh Genetics Limited	Watmind 2019 nCoV novel coronavirus antibody detection reagent	-	-	10 μL of serum or plasma; 20 μL of fingertip blood or whole blood	15
Getein Biotech, Inc.	One Step Test for Novel Coronavirus (2019-nCoV) IgM/IgG Antibody	94.1%	95.1%	10 μL of serum or plasma; 20 μL of fingertip blood or whole blood	10–20
Goldsite Diagnostics Inc.	SARS-CoV-2 IgG/IgM Kit	-	-	30 μL of whole blood	12
Hangzhou Biotest Biotech	COVID-19 IgG/IgM Rapid Test Cassette	IgM 91.8% IgG 100%	IgM 99.2% IgG 99.5%	10 μL of whole blood, serum or plasma	10
Hunan Lituo Biotechnology	COVID-19 IgG/IgM Detection Kit	-	-	-	15
InTec Products, Inc.	Rapid SARS-CoV-2 Antibody Test IgG or Ig Mor IgG/IgM	94.4%	98%	10 μL of sample	15–20
Liming Bio-Products Co., Ltd.	COVID-19 IgG/IgM Combo Rapid Test Device	IgG 93.1% IgM 64.7%	IgG 100% IgM 100%	10 μL of serum or plasma; 20 μL of whole blood	15
Livzon Diagnostic	Diagnostic Kit for IgM/IgG Antibody to Coronavirus (SARSCoV-2)	90.6%	99.2%	10 μL of serum or plasma; 20 μL of whole blood	15
nal von minden GmbH	NADAL^®^ COVID-19 IgG/IgM Test	94.1%	99.2%	10 μL of whole blood, serum or plasma	10
Nanjing Vazyme Medical Tech.	2019-nCoV IgG/IgM Detection Kit	91.54%	97.02%	20 μL of whole blood, serum or plasma	15
PRIMA Lab S.A.	PRIMA COVID-19 IgG/IgM Rapid Test (For Professional Use)	-	-	10 μL of serum or plasma; 20 μL of fingertip blood or whole blood	20
Sugentech, Inc	SGTi-flex COVID-19 IgM/IgG	90%-92%	96%-98%	10 μL whole blood	10
Sensing Self,	COVID-19 Rapid IgG/IgM combined Antibody assay	IgM 92% IgG 100%	IgM 97.58% IgG 99.31%	20 μL of fingertip blood or whole blood	10
Xiamen AmonMed Biotechnology	COVID-19 IgM/IgG test kit	IgM 78.43% IgG 84.31%	IgM 98.40% IgG 99.20%	-	15
Coris BioConcept	COVID-19 Ag Respi-Strip	60%	98–100%	100 μL extract	15
RapiGEN, Inc.	BIOCREDIT COVID-19 Ag	89.4	98%	90–150 μL extract	5–8
SD BIOSENSOR,	STANDARD Q COVID-19 Ag Test	84%	100%	10 μL extract	15–30
VivaChek Laboratories,	VivaDiagTM COVID-19 IgM/IgG Rapid Test	100%	IgM and IgG: 97.1%	10 μL of whole blood, serum or plasma	15
Qingdao Hightop Biotech	SARS-CoV-2 IgM/IgG Antibody Rapid Test	IgG 93% IgM 82%	IgG 97.5% IgM 96%	10 μL of serum or plasma 20 μL of whole blood	15–20
Novazym	Wuhan Coronavirus Rapid Test (2019-nCoV, COVID-19) IgG/IgM	IgG 91.8% IgM 95.7%	IgG 96.4% IgM 97.3%	5 μL of serum or plasma 10 μL of whole blood	15

**Table 8:** The descriptive characteristics of available serological immunodiagnostic COVID-19 tests and based on antibody-antigen and specimen (updated 13 June 2020, online sources).

Test Type	Developer	Test	Molecule	Materials	Status
**Immunochromatographic**	AccuBioTech Co. Ltd.	Accu-Tell COVID-19 IgG/IgM Rapid Test Cassette	IgG or IgM or both	Whole blood/serum/plasma	CE
	BIOMAXIMA S.A.	2019-nCoV IgG/IgM Rapid Test Cassette			CE-IVD
	BioMedomics, Inc	COVID-19 IgM-IgG Dual Antibody Rapid Test			CE-IVD; India
	Cellex Inc.	Cellex qSARS-CoV-2 IgG/IgM Cassette Rapid Test			CE-IVD; USA; Australia; Brazil
	Changsha Sinocare Inc.	SARS-CoV-2 Antibody Test Strip (Colloidal Gold Method)			CE-IVD
	Edinburgh Genetics Limited	Watmind 2019 nCoV novel coronavirus antibody detection reagent			CE-IVD
	Getein Biotech, Inc.	One Step Test for Novel Coronavirus (2019-nCoV) IgM/IgG Antibody			CE
	Goldsite Diagnostics Inc.	SARS-CoV-2 IgG/IgM Kit			-
	Hunan Lituo Biotechnology	COVID-19 IgG/IgM Detection Kit			-
	Innovita Biological Technology	2019-nCoV Ab Test (Colloidal Gold) IgM/IgG			CE-IVD; China, Brazil
	InTec Products, Inc.	Rapid SARS-CoV-2 Antibody Test IgG or Ig Mor IgG/IgM			CE-IVD
	Liming Bio-Products Co., Ltd.	COVID-19 IgG/IgM Combo Rapid Test Device			-
	nal von minden GmbH	NADAL^®^ COVID-19 IgG/IgM Test			-
	PRIMA Lab S.A.	PRIMA COVID-19 IgG/IgM Rapid Test (For Professional Use)			CE
	Dynamiker Biotechnology (Tianjin) Co., Ltd.	2019 nCOV IgG/IgM Rapid Test			CE-IVD
	Coris BioConcept	COVID-19 Ag Respi-Strip	Antigen	Nasopharyngeal secretions/swab	CE-IVD
	RapiGEN, Inc.	BIOCREDIT COVID-19 Ag			CE-IVD
	SD BIOSENSOR, Inc.	STANDARD Q COVID-19 Ag Test			CE-IVD, Brasil
**ELISA**	DRG International, Inc.	COVID-19 lgG, EIA-6146	IgG	Serum	-
	Epitope Diagnostics, Inc.	EDI™ Novel Coronavirus COVID-19 IgG ELISA Kit			CE-IVD
	EUROIMMUN AG	Anti-SARS-CoV-2 ELISA (IgG)			CE-IVD; Brazil; USA
	DRG International, Inc.	COVID-19 lgM, EIA-6147	IgM		-
	Epitope Diagnostics, Inc.	EDI™ Novel Coronavirus COVID-19 IgM ELISA Kit			CE-IVD
	EUROIMMUN AG	Anti-SARS-CoV-2 ELISA (IgA)	IgA		CE-IVD; Brazil
**immunofluorescent**	SD BIOSENSOR, Inc.	STANDARD F COVID-19 Ag FIA	Antigen	Nasopharyngeal swab	CE-IVD, Brasil
	Shenzhen Bioeasy Biotechnology Co., Ltd.	Bioeasy 2019-nCoV Ag Fluorescence Rapid Test Kit			CE-IVD
	Mokobio Biotechnology R&D Center	SARS-CoV-2 IgM & IgG Quantum Dot Immunoassay	IgG/IgM	serum, plasma, whole blood	-
**Proteome Microarray**	PEPperPRINT GmbH	PEPperCHIP^®^ SARS-CoV-2 Proteome Microarray (manual)	Proteome	Serum	-
